# Conference Abstracts: European Academy of Nursing Science Summer Conference 2019: Lisbon, Portugal. 10-11 July 2019

**DOI:** 10.1186/s12912-019-0370-y

**Published:** 2019-12-05

**The European Academy of Nursing Science (EANS)**

**Summer Conference 2019**

**Nursing at the centre of innovation and technology:**

**time for reflection on the challenges and opportunities**

**Book of Abstracts**

Escola Superior de Enfermagem de Lisboa (ESEL), Lisbon, Portugal

10^th^ - 11^th^ July 2019

Edited by Riitta Suhonen, Piia Riis Olsen and Sascha Köpke

**http://european-academy-of-nursing-science.com/**

## SPEAKER PRESENTATIONS

### S1 Feasibility of an intervention program to support families’ expressive functioning when a family member is depressed

#### Carmo Gouveia^1^, Eydis Sveinbjarnardottir^2^, Adriana Henriques^3^

##### ^1^Higher School of Health, University of Madeira, Funchal, Portugal; ^2^School of Health Sciences, University of Akureyri, Akureyri, Iceland; ^3^Lisbon Higher School of Nursing, University of Lisbon, Lisbon, Portugal

###### **Correspondence:** Carmo Gouveia (carmo.gouveia@gmail.com)

**Background**

Depression is a serious/disabling disease that affects family expressive functioning. Based on the Calgary Family Assessment and Intervention Models this study aimed to access the feasibility of an intervention program to promote family expressive functioning in depression.

**Materials and Methods**

An uncontrolled follow-up cohort study was accomplished using mixed methods. A non-random sample of 11 families (23 participants) received six 60 minutes’ sessions from mental health nurses. Assessment tools included 3 monitoring focus groups with mental health nurses, QFEF, QPSF and IACLIDE application and final semi-structured interviews with nurses and families. Data analysis was carried out through IBM SPSS Statistics (v.24) and NVIVO11 programs.

**Results**

The families reported better interaction patterns and a new understanding of the disease. The nurses identified new therapeutic intervention competences. Family expressive functioning and perceived support scores improved and depressed members’ depression levels decreased. All participants recognized the intervention benefits and suggested its continuity.

**Conclusions**

The intervention is feasible and acceptable by nurses and families and presents itself as a useful, organizing and guiding tool for mental health practice with families.

Funding FCT Grant NºSFRH/BD/113061/2015.

**References**

Wright,M.L. & Leahey,M.(2013). Nurses and Families: A guide to family assessment and intervention. (6ªed.). Philadelphia: Davis Company.

### S2 To test the feasibility of the psychoeducational intervention programme: the oncological disease in our lives, according to the methodology of complex interventions

#### Otília Barreto^1,2^, Adriana Henriques^3^

##### ^1^Regional Health Service of the Autonomous Region of Madeira, Portugal; ^2^University of Lisbon and School of Nursing of Lisbon, Lisbon, Portugal; ^3^Nursing School of Lisbon, Lisbon, Portugal

###### **Correspondence:** Otília Barreto (maria.barreto@campus.esel.pt)

**Background**

The person and family to whom an oncological disease is diagnosed present changes in their emotional state. Some people require the specialized intervention of health professionals in order to adopt effective strategies to deal with the disease. The aim was to test the feasibility of the psychoeducational intervention program: the oncological disease in our lives, according to the methodology of Complex Interventions.

**Materials and methods**

The development of the psychoeducational program followed the guidelines recommended by the Medical Research Council. The program consisted of 6 people (patients and their families). To these participants the Focus Group technique was applied in order to know their perception about the program.

**Results**

The findings show that the program is relevant and responds to the needs of the participants. All participants would recommend the program to people who are in a similar situation.

**Conclusions**

The psychoeducational intervention program: the oncological diseases in our lives, is a specific intervention and an innovative tool for clinical practice of nurses who develop their professional activity in this area.

### S3 Co-producing and evaluating an innovative eHealth intervention for family carers of people with psychosis – the EFFIP Project (E-support for Families & Friends of Individuals affected by Psychosis)

#### Jacqueline Sin^1^, Claire Henderson^2^, Luke Woodham^3^, Aurora Sese^3^, Dominique Spence-Polin^3^, Steve Gillard^1^

##### ^1^Population Health Research Institute, St George’s, University of London, London, UK; ^2^Health Service & Population Research Department, Institute of Psychiatry, Psychology & Neuroscience, King’s College London, London, UK; ^3^Institute of Medical and Biomedical Education, St George’s, University of London, London, UK

###### **Correspondence:** Jacqueline Sin (jasin@sgul.ac.uk)

**Background**

eHealth interventions are growing popular in managing a wide range of public mental health issues. Nonetheless, eHealth interventions are also well-known to be inferior in terms of sustaining engagement with their end-users. The EFFIP project is a five-year research programme aiming to develop and evaluate a multi-component eHealth intervention for carers of people with psychosis.

**Methods**

We adapted the MRC complex intervention framework to incorporate significant participatory research activities involving key stakeholders and carers as end-users to co-design and co-produce the eHealth intervention, using an agile build process. Further public involvement activities are integral to the ongoing project oversight and management of the evaluative study.

**Results**

Co-production work helped optimise the intervention design. Further, we conducted a usability study on the prototype involving carers to test the delta-build. These have led to the co-production of an eHealth intervention "COPe-support (Cares fOr People with Psychosis e-support)" - providing information and psychosocial support for carers through the internet, promoting flexible access and individualized choices.

**Conclusions**

We believe the co-production work has optimised the intervention design and usability fitting the end-users’ needs and usage pattern in the real world. COPe-support is currently being evaluated through an England-wide RCT (http://www.isrctn.com/ISRCTN89563420)

### S4 Health promotion in schools: community and public health nurses’ challenges

#### Andreia Costa^1^, Jorge Almeida^2^, Fátima Ramalho^2^

##### ^1^Escola Superior de Enfermagem de Lisboa, Instituto de Saúde Ambiental (ISAMB), University of Lisbon, Lisbon, Portugal; ^2^ACES, Amadora, Portugal

###### **Correspondence:** Andreia Costa (andreia.costa@esel.pt)

**Background**

Health promotion to young people in schools, sustained in the practice based on evidence, is one of the main roles of the Community and Public Health Nurses. Portugal digital market registered great investments including digital health literacy.

The aim of this study was to promote knowledge that leads to the prevention of alcohol consumption in adolescents.

**Materials and methods**

Systematic review of the literature from the Scoping Review type, according to the Joanna Briggs Institute protocol and Health Planning methodology.

**Results**

The data analysis before the Community Intervention revealed a lack of knowledge on the several aspects of alcohol. With the Community Intervention this deficit in knowledge decreased. Although this positive result it seems to be a lack of digital approaches in health promotion, considering that 67.3 % of Portuguese are Internet Users, lower than European Union average, and the new Action Plan for Health Literacy, were adolescents are a digital generation, new digital approaches should be considered.

**Conclusions**

The positive results suggest the acquisition of knowledge about alcohol. The evaluation reveal that adolescents considered the Community Intervention an important mean to acquire knowledge but digital approaches should be considered in order to promote health literacy in schools.

### S5 Community health and public health nurse: innovation, realities and challenges

#### Andreia Costa, Carmen Cunha, Maria Adriana Henriques

##### Escola Superior de Enfermagem de Lisboa, University of Lisbon, Lisbon, Portugal.

###### **Correspondence:** Andreia Costa (andreia.costa@esel.pt)

**Background**

Facing challenges and new opportunities for public health, nursing also has to question itself. The aim was to analyse competences and interventions of community and public health nurses in the context of technological innovation.

**Materials and Methods**

Systematic Review of Literature of the last ten years, following an Equator Network protocol.

**Results**

The qualitative analysis showed that skills and interventions are close. We highlight the purpose of developing a nursing model that frames nurses' practice in the community, that focuses on three diagnostic dimensions: participation, leadership and community process, as well as the case of a new public health nursing training program, specifically prepared to be available on the internet, with the aim of establishing nurses in this area and providing job satisfaction. An innovative approach to improving public health nursing competencies, through the promotion of intra-professional and community collaboration and the principles of service-learning emerge due to new information systems who support clinical registries and epidemiological surveillance.

**Conclusions**

The variety of interventions adds the multiplicity of settings, although the main focus of most public health nurses is vaccination. The need for development of models with competencies and interventions of the community and public health nurse will implement collaboration, adapts to new technologies.

### S6 Emerging technologies in perioperative care: a literature review

#### Petros Kolovos^1^, Aggeliki Athanasopoulou^2^, Styliani Tziaferi^1^

##### ^1^Department of Nursing, University of Peloponnese, Tripoli, Greece; ^2^General Hospital of Messinia, Kalamata, Greece;

###### **Correspondence:** Styliani Tziaferi (stylianitziaferi216@gmail.com)

**Background**

A growing number of technological innovations have been integrated into clinical practice. The adoption of technologies in health care settings is linked with improvements in the delivery of high quality and safety of patient care. Moreover, communication exchange and collaboration among personnel is facilitated. The aim was to investigate the contemporary literature on the emerging technologies in perioperative setting.

**Materials and methods**

A literature review was conducted in PubMed, Scopus and Google Scholar with the keywords: ‘emerging technologies, ‘digital innovations’, ‘monitoring techniques’, ‘surgical care’ and ‘perioperative care’. Articles published in the last 10 years in English language were included. Data were analyzed and synthesized.

**Results**

The perioperative setting is a complex and technologically advanced environment. There is evidence that the emerging technologies have been implemented across the perioperative pathway: preoperatively: digital interventions to improve management of risk factors and enhance patient adherence, intraoperatively: non-invasive technologies and sensors, decision support tools, electronic checklists, and postoperatively: devices for monitoring physical activity and mobilization, pain management and for detecting complications.

**Conclusions**

With the support of emerging technologies an individualized and safe care is ensured. Relevant further nursing research of evidence‐based practice is encouraged in Europe to optimize patient outcomes.

**References**

Haahr-Raunkjær C, Meyhoff CS, Sørensen HBD, Olsen RM, Aasvang EK. Technological aided assessment of the acutely ill patient–The case of postoperative complications. European Journal of Internal Medicine. 2017;45:41-45.

Michard F, Gan TJ, Kehlet H. Digital innovations and emerging technologies for enhanced recovery programmes. BJA: British Journal of Anaesthesia. 2017;119(1):31-39.

Stabile M, Cooper L. The evolving role of information technology in perioperative patient safety. Canadian Journal of Anesthesia/Journal Canadien d'Anesthésie. 2013;60(2):119-126.

### S7 Future innovations in nursing and health care and challenges to nursing education

#### Natalja Fatkulina (Istomina)^1^ (natalja.fatkulina@mf.vu.lt), Karen Amlaev^2^

##### ^1^Faculty of Medicine, Vilnius University, Vilnius, Lithuania; ^2^ Stavropol State Medical University, Stavropol, Russia

**Background**

The development of new medical technologies will increase life expectancy by 30 years and reduce the death rate from cardiovascular diseases by 47%, malignant formations by 30%, and HIV infections and associated pathologies by 40%. The current education system prepares health specialists for past realities, not future ones. The competence of nurse should be developed in innovative way.

**Materials and Methods**

A literature review was done 20–24 April 2019 with the following search words: medical technologies, nursing technologies, innovations in education, and new professions in health care. One hundred fifty-one articles were found. After an evaluation based on the abstracts, seven articles were analysed.

**Results**

The challenges of the future influence the development of new roles for nurses. Those challenges include the creation of mobile diagnostic devices for self-diagnosis and early diagnosis, development and management of high-tech medical equipment, genome programming for specified parameters, establishment of personal insurance programmes according to a patient’s genetic card, and ensuring communication between research, nursing, diagnosis and treatment.

**Conclusions**

The most important learning outcomes for nurses that will influence the future include the following: 1) knowledge, skills, and competencies; 2) environment, social capital, and networks; 3) personal transformation and inspiration.

### S8 Innovative application of a MOOC (Massive Open Online Course) within the Lydia Osteoporosis Project (LOP 3), an action research, process evaluation and implementation project

#### Margaret A Coulter Smith, Claire Pearson, Denny Roberts

##### Division of Nursing, Queen Margaret University, Edinburgh, United Kingdom

###### **Correspondence:** Margaret A Coulter Smith (msmith1@qmu.ac.uk)

**Background**

This presentation focuses on the design, implementation and evaluation of a Massive Open Online Couse (MOOC), a complex online learning intervention within an action research, process evaluation and implementation project (The Lydia Osteoporosis Project 3).

**Materials and Methods**

The action research phase includes adaptation of a ‘Caring for My Bones’ online course for community settings. This ‘complex learning’ initiative involves learners’ assimilating knowledge, skills and attitudes about osteoporosis, fracture risk, then bringing together various skills components related to promoting mobility, safe and person-centred moving and handling, and ultimately transferring this learning in their work settings (van Merriënboer & Kirschner 2013). Research participants include health and social care staff, managers and patients/ residents. Ethics and Research Governance approvals were obtained for all research sites.

**Results**

The MOOC launched January 2019. 160+ participants registered. Course duration was extended from 8 to 12 weeks. Formative comments and summative evaluations were very positive.

**Conclusions**

A well designed online learning intervention within action research, offers a way to engage staff and stakeholders in complex learning with potential for transforming practice.

**References**

van Merriënboer J J G, Kirschener P A 2013 Ten Steps To Complex Learning. 2nd ed. Routledge, London.

### S9 New technologies in the practical classes of the nursing course: students' opinions

#### Cristina Barroso, Maria José Lumini

##### Oporto Nursing School, Porto, Portugal

###### **Correspondence:** Cristina Barroso (cristinabarroso@esenf.pt)

**Background**

The growth of technological knowledge has enabled the development of digital simulators in education, promoting the understanding and integration of nursing knowledge. However, during students’ nursing practice the development of instrumental skills is a key factor. The aim of this study was to identify the technologies able to assist nursing students in the development of their instrumental skills.

**Materials and Methods**

This is an exploratory, descriptive and qualitative study. The data were collected from nursing students using the focus group method after the completion of practical classes of the second semester curricular unit of the nursing course. The sample comprised 39 participants, divided into four focal groups.

**Results**

The development of students’ instrumental skills requires a detailed description of the procedures, explaining each step, supported by the use of photos and videos.

**Conclusions**

Considering the findings of this study and the specific characteristics of the students involved, the design of an App would help students developing their instrumental skills.

**Reference**

Kim, J., Park, J. H., & Shin, S. (2016). Effectiveness of simulation-based nursing education depending on fidelity: a meta-analysis. BMC Medical Education, 16, 152. doi:10.1186/s12909-016-0672-7.

### S10 Contributions of electronic health records in nursing knowledge representation: diagnoses centered on Orem's universal self-care requisites

#### Carmen Queirós^1,2,3,4^, Maria Antónia Taveira da Cruz Paiva e Silva^2^

##### ^1^Instituto Ciências Biomédicas Abel Salazar, University of Porto, Porto, Portugal; ^2^Centre for Information Systems and ICNP® Research and Development, Porto Nursing School, Porto, Portugal; ^3^Centro Hospitalar Universitário do Porto, EPE, Porto, Portugal; ^4^ Health Sciences Research Unit: Nursing (UICISA: E), Coimbra Nursing School (ESEnfC), Coimbra, Portugal

###### **Correspondence:** Carmen Queirós (carmenqueiros@gmail.com)

**Background**

Self-care is a central concept in the nursing discipline. Nursing knowledge representation in clinical settings is anchored in nursing care documentation. Portugal stands out among the countries of the European Union (EU) with greater use of electronic health records (EHR) based on a classified professional language - International Classification of Nursing Practice (ICNP®). The aim of the study was to identify nursing diagnoses centred on Orem's universal self-care requisites documented by nurses in Portugal.

**Materials and Methods**

1) All nursing documentation customized in Portuguese public EHR was submitted to Bardin's content analysis 2) An expert panel explored the results from content analysis

**Results**

3782 nursing diagnoses centred on Orem's universal self-care requisites were identified on EHR. 112 nursing diagnoses were considered clinically meaningful after content analysis by the expert panel.

**Conclusions**

Despite the widespread use of EHR based on ICNP® in Portugal, nursing diagnoses documentation faces challenges due to the use of different terms and concepts to represent the same diagnoses. Nursing knowledge representation into clinical data models which can be use in the backend of EHR will improve nursing decision-making quality and consequently nursing care.

**Acknowledgements**

Centre for Information Systems and ICNP® Research and Development of Porto Nursing School

### S11 Knowledge representation in nursing health records related to self-management of medication

#### Inês Cruz, Fernanda Bastos, Filipe Pereira, Ernesto Jorge Morais, Fernando Oliveira, Paula Prata, Abel Paiva

##### School of Porto, Porto, Portugal

###### **Correspondence:** Inês Cruz (inescruz@esenf.pt)

**Background**

Clinical decision-making process plays an important role in the quality of care that nurses provide to patients. In this process nurses collect data, identify diagnoses and prescribe interventions to minimize, prevent or resolve the nursing diagnoses. The aim of the study was to identify data, diagnoses and interventions related to self-management of the medication regimen.

**Materials and Methods**

Content analysis of the documentation customized in national nursing records (SAPE® and Sclínico®). Literature review to identify data, diagnoses and interventions related to self-management of medication regimen.

**Results**

Five relevant data were identified to judge about the impaired self-management of the medication regimen. 13 diagnosis and 49 nursing interventions were identified. The Interventions intended totally or partly to replace the person. When the problem lies in how the person manages the medication, other data, diagnoses and interventions have emerged related to knowledge, ability, awareness, self-efficacy and meanings.

**Conclusions**

In the decision-making process, nurses must not only identify patients' problems and implement interventions that minimize or resolve them, but also proceed to the identification of each person's “readiness to enhance” self-management skills. This study contributes to the formalization of the disciplinary knowledge in this area and helps in clinical decision-making process.

### S12 Exploring nursing records using ICNP® in long-term care

#### Maria Joana Campos, Abel Paiva e Silva

##### Nursing School of Porto, Porto, Portugal

###### **Correspondence:** Maria Joana Campos (joana@esenf.pt)

**Background**

It is important to know more about the care that professional nurses perform in long-term care at home. The aim of the study was to identify types and frequencies of diagnosis, interventions and outcomes in this context.

**Materials and Methods**

Data was extracted from the Nursing Information System and transferred to a new database for analysis. ICNP ® was used as referential terminology in data analysis

**Results**

The nursing records were from 191 patients/cases. The two most frequent phenomena were related to tissue integrity and Self Care. Related to tissue integrity, risk of pressure ulcer was on top. The most relevant self-care diagnosis was related to self-care deficit, like ability to perform self-care (transfer, walking using device, self-turning). 192798 interventions were recorded from 1515 syntaxes. By type of action ICNP**®**, determining have a global implementation of 37.75%, followed by the type of action informing (20.87%), attending (18.99%), performing (11.80%) and finally those interventions using the type of action managing (10.59%).

**Conclusions**

Data and information are the key elements to improve quality of nursing care. The most relevant outcomes were related to improving ability to self-care. The study reflects nursing practice in Europe.

### S13 Is technology a hot topic? Analysis of nursing doctoral thesis titles in Portugal

#### Florinda Galinha de Sá, Sonia Ferrão, Maria Antonia Rebelo Botelho, Adriana Henriques

##### Superior de Enfermagem de Lisboa, Lisbon, Portugal

###### **Correspondence:** Florinda Galinha de Sá (fgalinha@esel.pt)

**Background**

Undertaken research reported in doctoral nursing thesis over time helps to identify and reflect on changing trends and evolution in nursing knowledge development. The aim of this study was to analyse the information of nursing doctoral thesis titles published in Portugal.

**Materials and Methods**

All doctoral theses completed in Nursing Doctoral Programmes in Portugal, up until 2018, available in the university's digital repositories were included. Thesis titles were appraised and classified into general study topics, participants, health category, setting and research methods. Data were analysed using descriptive statistics.

**Results**

A total of 279 nursing doctoral theses were included. A significant number of thesis titles reported vague information regarding study subject, setting, participants and research methods, despite numerous long titles (more than 16 words). The most often researched topics reported were related to elderly care, family care, and informal caregivers. Only seven thesis titles addressed technology and most of them were published in 2016 and 2017.

**Conclusions**

Health technology is a recent topic in nursing research in doctoral theses in Portugal.

Elderly care, family care, and informal caregivers are common research topics, highlighting key

21^st^ century health issues.

### S14 Computerized decision support: a valuable means to support nurses' clinical judgement and decision making?

#### Theresa Thoma-Lürken, Michel HC Bleijlevens, Jan PH Hamers

##### Department of Health Services Research, Maastricht University, Maastricht, The Netherlands

###### **Correspondence:** Theresa Thoma-Lürken (T.thoma@maastrichtuniversity.nl )

**Background**

Nurses working in community-based dementia care are increasingly challenged with complex clinical judgments and decision-making. Computerized decision support tools may be promising means to support nurses with these tasks. The aim was to develop and evaluate a decision support App for nurses in community-based dementia care.

**Materials and Methods**

A multi-method approach was used, combing usability studies, interviews, focus groups and a laboratory experiment. Nurses as end users were involved in all phases.

**Results**

A usable decision support App was developed, aiming to facilitate a structured problem assessment with regard to problems preventing people with dementia from living at home and to provide an overview of possible solutions. The efficacy of the App in terms of improved decision-making has not been proven, however nurses emphasized its potential added value for their work.

**Conclusions**

The decision support App is perceived as a valuable mean to support nurses’ clinical judgment and decision-making in community-based dementia care. However, more research is needed to evaluate its efficacy. The role of community nursing in health care is rapidly increasing while nurses’ decision-making is getting more complex. Technology can support nurses in making adequate decisions and improve their clinical judgement.

**References**

Thoma-Lürken, T., Bleijlevens, M. H. C., Lexis, M. A. S., & Hamers, J. P. H. (2018). Evaluation of a decision support app for nurses and case managers to facilitate aging in place of people with dementia. A randomized controlled laboratory experiment. *Geriatric nursing , 39*(6), 653-662. Retrieved from https://www.ncbi.nlm.nih.gov/pubmed/29858041. doi:10.1016/j.gerinurse.2018.04.019

Thoma-Lürken, T., Lexis, M. A. S., Bleijlevens, M. H. C., & Hamers, J. P. H. (2018). Development and usability of a decision support App for nurses to facilitate aging in place of people with dementia. *Applied Nursing Research, 42*, 35-44. Retrieved from http://www.sciencedirect.com/science/article/pii/S0897189717306079. doi:https://doi.org/10.1016/j.apnr.2018.04.008

Thoma-Lürken, T., Lexis, M. A. S., Bleijlevens, M. H. C., & Hamers, J. P. H. (2019). Perceived added value of a decision support App for formal caregivers in community-based dementia care. *Journal of Clinical Nursing, 28*(1-2), 173-181. Retrieved from https://www.ncbi.nlm.nih.gov/pubmed/30091499. doi:10.1111/jocn.14647

### S15 Validation of an educational programme for self-management, on adolescents with spina bifida

#### Isabel Costa Malheiro^1^, Filomena Gaspar^1^, Luisa Barros^2^

##### ^1^Escola Superior de Enfermagem de Lisboa, Lisbon, Portugal; ^2^ Psychology Faculty, Lisbon University, Lisbon, Portugal

###### **Correspondence:** Isabel Costa Malheiro (mmalheiro@esel.pt)

**Background**

The number of children with Spina Bifida (SB) who survived reaching adulthood increased significantly and their adolescence transition is a major concern. The aim of this study was to validate the effectiveness of an educational program that promotes functional independence (Daily Living Activities) and self-management competencies.

**Materials and Methods**

139 adolescents with SB (10-18 years) performed the Program. With a before (T1) and after (T2 and follow-up T3) design, the analysis was performed using the program IBM SPSS Statistics 22.

**Results**

The results revealed that the adolescents made a significant gain on functionality, cognitive and motor domains with moderate to high effect sizes observed. The program produced better effects on young people aged between 10 and 12 years without previous experience on camps, regardless the gender, level of injury, presence of hydrocephalus or the type of auxiliary gait devices they use.

**Conclusions**

The program had greatest impact in the motor domain of the functionality (self-care, elimination, transfers), which remains six months later. This program support that the experience improves the self-management competences and the functional independence motor dimension of youth with SB and, suggest that the effectiveness of the program is valid.

### S16 TALKING TIME: A pilot randomized controlled trial investigating social support for informal caregivers via the telephone

#### Martin Nikolaus Dichter^1,2^, Bernd Albers^1,2^, Diana Trutschel^2^, Armin Michael Ströbel^1^, Swantje Seismann-Petersen^1^, Katharina Wermke^3^, Margareta Halek^1,2^, Martin Berwig^1,2,3^

##### ^1^German Center for Neurodegenerative Diseases (DZNE), Witten, Germany; ^2^School of Nursing Science, Witten/Herdecke University, Witten, Germany; ^3^Clinic and Policlinic for Psychiatry and Psychotherapy, Leipzig University, Leipzig, Germany

###### **Correspondence:** Martin Nikolaus Dichter (Martin.Dichter@dzne.de)

**Background**

Caring for people with dementia (PwD) at home requires considerable time, organization and commitment. Therefore, informal caregivers (ICs) of PwD are often overburdened.

**Materials and Methods**

This study was a MRC framework phase two randomized controlled trial. The intervention consisted of a preliminary talk, information booklet, six structured telephone-based support group meetings and a structured written self-evaluation of each support group meeting. The control participants performed their usual individual self-organized care.

**Results**

Thirty-eight ICs and their relatives were included and allocated to the intervention or control groups (n = 19 each). After three months, the Talking Time intervention group demonstrated an increase in the SF-12 scores (primary outcome), whereas the scores decreased in the control group. However, the standardized effect size of 1.65 (95% Confidence Interval: -0.44 – 3.75) was not significant (p = 0.42). Additionally, the secondary outcomes demonstrated no significant results. The differences between the groups in most outcomes were in the expected direction.

**Conclusions**

The Talking Time intervention is feasible and shows nonsignificant promising results with regard to the self-rated psychological HRQoL. After further adjustment, the intervention needs to be evaluated in a full trial.

Trial Registration

Clinical Trials: NCT02806583

### S17 Elders with type 2 diabetes and health professionals’ perspectives on the pre-requisites of a virtual assistant prototype to support self-care (VASelfCare)

#### Isa Félix^1^, Adriana Henriques^1^, Afonso Cavaco^2^, Anabela Mendes^1^, Ana Paula Cláudio^3^, Beatriz Jesus^1^, Isabel Costa e Silva^1^, João Balsa^3^, Maria Beatriz Carmo^3^, Maria Guedes^1^, Nuno Pimenta^4^, Pedro Alves^3^, Pedro Neves^3^, Rita Gutierrez^1^, Mara Guerreiro^1^

##### ^1^Escola Superior de Enfermagem de Lisboa, Lisbon, Portugal; ^2^ Faculty of Pharmacy, Lisbon University, Lisbon, Portugal; ^3^Faculty of Sciences, Lisbon University, Lisbon; ^4^Sport Sciences School of Rio Maior – IP Santarém, Rio Maior, Portugal

###### **Correspondence:** Mara Guerreiro (mara.guerreiro@esel.pt)

**Background**

In Europe, type 2 Diabetes (T2D) affects 35.9% of people over 65 years old. Digital interventions have successfully been used to enhance self-care behaviours. This study is part of the development of a multi-behaviour intervention via a mobile application prototype with an anthropomorphic virtual assistant (VA). The aim was to describe the perspectives of elders with T2D and health professionals on the prototype pre-requisites.

**Materials and Methods**

Contact with the first prototype was used to elicit informed opinions from healthcare professionals (n=19) and elders with T2D (n=9) on pre-requisites. Data were collected through a questionnaire with closed and open-ended questions administered by an interviewer in five primary care units. Ethical approval was granted.

**Results**

Overall, the most frequent score was 4 in a 1 to 5 Likert scale (46,9%). The only significant difference between responses of the two groups concerned the VA facial expression (mean value for professionals 3.37 vs patients 4.33, t=3.00, p=0.006). In general, similarities about the perspectives on content and application features emerged from textual data.

**Conclusions**

Results were overall positive and highlighted opportunities for improvement, which will be actioned and further tested in the same groups. Relevance for nursing science in Europe: This study illustrates the co-production of an application prototype with nurses and final users.

### S18 Implementing behaviour change techniques for healthy eating in a virtual assistant prototype to support self-care in older people with type 2 diabetes (VASelfCare)

#### Isa Félix^1^, José Camolas^2^, Adriana Henriques^1^, Afonso Cavaco^3^, Anabela Mendes^1^, Ana Guerreiro^4^, Ana Paula Cláudio^5^, Catarina Costa^4^, João Balsa^5^, Madalena Carvalho^4^, Maria Beatriz Carmo^5^, Pedro Alves^5^, Pedro Neves^5^, Mara P. Guerreiro^1^

##### ^1^Escola Superior de Enfermagem de Lisboa, Lisbon, Portugal; ^2^Serviço de Endocrinologia-CHULN/Laboratório de Nutrição-FMUL, Lisbon, Portugal ^3^Faculty of Pharmacy, Lisbon University, Lisbon, Portugal; ^4^Lisbon Nursing School, Lisbon, Portugal; ^5^Faculty of Sciences, Lisbon University, Lisbon

###### **Correspondence:** Mara P. Guerreiro (mara.guerreiro@esel.pt)

**Background**

Healthy eating is linked to the control of type 2 diabetes (T2D). This study is part of a multi-behavior intervention via a mobile application prototype with an anthropomorphic virtual assistant (VA), guided by the MRC framework for complex interventions. The aim of the study was to describe evidence-based diet behaviour change techniques (BCTs) in T2D and their operationalisation in the prototype.

**Materials and Methods**

Literature review on effective dietary BCTs in T2D. BCTs were then selected for operationalisation in the eight diet topics covered by the prototype, through multidisciplinary consensus discussion.

**Results**

Two meta-analysis were retrieved. Nine effective BCTs were identified (BCTTv1 taxonomy), of which 7 were selected. For example, “Instruction on how to perform a behavior” was implemented in the “Reading the labels” topic by having the VA explaining verbally how to read food labels (e.g. fat, sugar and salt content). For “Demonstration of the behaviour” in the “Portion control” topic the VA resorts to a virtual plate model.

**Conclusions**

Seven effective diet BCTs were implemented in the prototype across different topics. A detailed description of BCTs implementation facilitates potential replication. BCTs are the active ingredients of this complex intervention; describing them may be useful for nurse scientists working in the field.

### S19 Cross cultural validation of the Iceland- Family Perceived Support Questionnaire (ICE-FPSQ) to European Portuguese

#### Carmo Gouveia^1^, Maria João Barreira Rodrigues^1^, João Carvalho Duarte^2^, Eydis Sveinbjarnardottir^3^, Adriana Henriques^4^

##### ^1^Higher School of Health, University of Madeira, Funchal, Portugal; ^2^Viseu Higher School of Health, Polytechnic Institute of Viseu, Viseu, Portugal; ^3^School of Health Sciences, University of Akureyri, Akureyri, Iceland; ^4^Lisbon Higher School of Nursing, University of Lisbon, Lisbon, Portugal

###### **Correspondence:** Carmo Gouveia (carmo.gouveia@gmail.com)

**Background**

Perceived benefits associated with health care and the effectiveness of nursing interventions requires suitable instruments that capture family member’s perceptions of cognitive and emotional support received from nurses. This study aimed to perform the linguistic and cultural adaptation of the Iceland-Family Perceived Support Questionnaire (ICE-FPSQ) to Portuguese and to determine its psychometric properties.

**Materials and Methods**

A translation/back-translation, cultural component analysis, external experts meeting and longitudinal screening of concepts and construct were accomplished. The pilot study was carried out in a sample of 119 members of Portuguese families with recent experience of depression. Validity and reliability were verified through IBM SPSS Statistics (v.24) and IBM SPSS AMOS (v24) programs.

**Results**

Exploratory factor analysis extracted two factors (65.164% of total variance). The temporal stability (0.68) and internal consistency (0.94) were good. Confirmatory factor analysis confirmed the original two-factor model and a good model fit.

**Conclusions**

The Portuguese version of ICE-FPSQ is reliable and valid with families affected by depression and can be applied in clinical and research contexts to Portuguese families.

Funding FCT Grant NºSFRH/BD/113061/2015.

**References**

Sveinbjarnardottir,E.K.;Svavarsdottir,E.K.;Hrafnkelsson,B.(2012).Psychometric development of the Iceland-Family Perceived Support Questionnaire. .Journal of Family Nursing,18(3),328-352

### S20 Digital transformation in caregiver family

#### Andreia Costa^1,2^ (andreia.costa@esel.pt)

##### ^1^Escola Superior de Enfermagem de Lisboa, Lisbon, Portugal; ^2^ISAMB, Instituto de Saúde Ambiental da Faculdade de Medicina de Lisboa, Lisbon, Portugal

**Background**

Digital transformation of health systems is happening, while ageing, prevalence of chronic diseases are challenges for health systems and societies where dependent people in self-care increase. Digital transformation can link health professionals to people in their homes. It’s essential to reflect on how digitization of health can support nurses’ interventions in caregivers’ families of dependent persons. The aim of the study was to explore the resources that caregivers’ families consider necessary to care for dependent people in self-care in their homes and the use of resources.

**Materials and Methods**

A quantitative descriptive survey. About 2500 families were studied.

**Results**

121 families included dependent persons where most of the required resource (e.g. bed transfer) utilization rates were less than 50%. The main reasons for not using the necessary resource were derived from knowledge deficits of families.

**Conclusions**

It is essential to develop models focused on the professional help that nurses can provide to families through guidance that allows them to use the resources most appropriate to the situation of dependency, promoting the patient’s autonomy whenever possible. Digital transformation must be an integrated element in healthcare systems offering relevant information to families to improve adequate resources in their everyday life.

### S21 Comparison of outcomes of various educational methods and cost-effectiveness in basic life support

#### Nikolaos Tsaloukidis^1^; Styliani Tziaferi^2^, Athina Lazakidou^1^

##### ^1^University of Peloponnese, Tripoli, Greece; ^2^University of Peloponnese, Sparta, Greece

###### **Correspondence:** Styliani Tziaferi (stylianitziaferi216@gmail.com)

**Background**

Comparison of educational results of different educational methods that can be used in mass adult education.

**Materials and Methods**

Educational programs on the subject of Basic Life Support. Setting: The research was conducted from October 2017 until March 2018. Participants were 90 healthcare professionals, out of whom 43 were trained by Distance Learning and 47 with Live Learning in Basic Life Support. The criteria studied were the knowledge and skills acquired by the trainees based on the different educational approach to Basic Life Support. In order to carry out the study, special training software for Basic Life Support was developed, focusing on the 2015 ERC guidelines that were adapted to the Moodle e-Learning Management System.

**Results**

The skills-based assessment of the trainees was realized with the use of the Resusci Anne QCPR manikin special model, utilizing the Resusci Anne Wireless Skill Reporter software, ver 2.0.0.14, by Laerdal. The results showed a QCPR score of 72.3% for Live Learning and 82.2% for Distance Education. Regarding the level of knowledge, the trainees responded to 20 Multiple Choice Questions, the results of which showed success in 74.3% in Live and 95.2% in Distance Learning.

The economic evaluation was based on the Legislative Framework and the economic situation in the Greek territory. The evaluation of the programs was based on the Cost-Effectiveness Analysis and the Incremental cost-effectiveness ratio, based on 25 trainees per training program / 2 programs per month, at a depth of five years. The cost of the educational programs, based on the above mentioned data, amounts to 29.92€ per person in Distance Learning, while in Live Learning the cost is 30.32€ per person. This cost tends to decline in both educational methods by increasing the number of trainees per program. The cost is set at 17.46€ for Distance and 27.65€ for Live Learning, with a number of 50 students per training program and the price differential widens

**Conclusions**

The cost of educational programs using Distance Learning is significantly lower, especially when the number of trainees is high.

## POSTER ABSTRACTS

### P1 Fundamentals of care in critical care nursing: back to basics and moving forward

#### Candida Durao, Helga Rafael Henriques

##### Escola Superior de Enfermagem de Lisboa, Lisbon, Portugal

###### **Correspondence:** Candida Durao (candida.durao@esel.pt)

**Background**

The fundamentals of care is not recognized as it should, resulting in patient safety threats, dehumanised care and poorer care outcomes. In critical care settings the challenge is to maintain humanity when using technology to promote a more in depth knowledge of clients. The technology becomes an extension of the nurses' hands, eyes, ears and other senses. The Portuguese Nursing Council established that critical care nursing competencies are developed in postgraduate or second cycle studies. At ESEL 150 nurses completed their master in critical care nursing. The aim was to highlight the fundamentals of care most valued by ESEL’s critical care nursing master students in their final reports.

**Materials and Methods**

Data were collected from the final reports of the student that completed the program until 2018. The Fundamentals of Care Framework guided the analysis.

**Results**

The relationship between the patient and the nurse was one of the core themes. The integration of care – Psychosocial, Physical and Relational - was the most valued dimensions.

**Conclusions**

To move forward in European critical care nursing is essential to be aware of the Fundamentals of care dimensions. Being technologically competent allows nurses to assess the patients including specific data that they could not obtain otherwise.

### P2 Leveraging eNursing to address the needs of older people with chronic diseases

#### Helga Rafael Henriques^1^, Emília Brito^1^, Andreia Costa^1^, Mara Guerreiro^1^, Luís Lapão^2^, Isabel Pereira^1^, Miriam Almeida^1^, Sara Luz^1^, Adriana Henriques^1^

##### ^1^ Escola Superior de Enfermagem de Lisboa, Lisbon, Portugal; ^2^ Global Health and Tropical Medicine, Instituto de Higiene e Medicina Tropical, Universidade Nova de Lisboa, Lisboa, Portugal

###### **Correspondence:** Helga Rafael Henriques (hrafael@esel.pt)

**Background**

Ageing and chronic diseases are increasing dramatically. Shortages in human resources and health care costs demand innovation by combining integrated nursing care with digital technologies. The aim was to describe a multidisciplinary research programme to develop and evaluate eNursing complex interventions in older people with chronic diseases.

**Materials and Methods**

We followed the next steps: 1) forming the multidisciplinary team across Institutions; 2) Deciding on the methodological approach; 3) Reviewing the literature; 4) Defining the nature, target and scope of the interventions in light of unmet needs.

**Results**

Seven researchers (nursing, pharmacy, public health and engineering) and four nursing doctoral students comprise the multidisciplinary research team. To ensure that eNursing interventions are clinically relevant and rigorous, the Medical Research Council (MRC) framework for complex interventions was chosen. Based on Design Science Research Methodology (DSRM), a digital platform for eNursing will be developed and implemented to support older people with chronic diseases, such as diabetes, cardiovascular diseases, chronic obstructive pulmonary disease and rheumatoid arthritis.

**Conclusions**

A research programme to address unmet needs of older people with chronic disease was set up. Next steps entail defining the components of the interventions and integrating the potential stakeholders. This will encompass health literacy and health status monitoring, addressing European patient-centeredness.

### P3 Promotion of self-care in patients with chronic illnesses; contributions of information and communication technologies (ICT)

#### Maria do Céu Sá^1,2^, Ana Sofia Campos Nabais^1^

##### ^1^Escola Superior de Enfermagem de Lisboa (ESEL), Lisbon, Portugal; ^2^UI&DE, Lisboa, Portugal

###### **Correspondence:** Maria do Céu Sá(ceu.sa@esel.pt)

**Background**

Chronic diseases are the leading causes of disability and morbidity that cause major health problems. Information and Communication Technologies (ICT) provide a framework for patient engagement and a new model of care that enables rapid response to the needs of the person / family. The promotion of self-care is one of the major challenges of health care, particularly for nurses, and technologies are increasingly used by patients to seek information and knowledge about their health status. The aim was to verify the extent to which ICT help patients with chronic diseases to promote their self-care and to manage their disease.

**Materials and Methods**

We performed a systematic literature review and the key-words used were: chronic disease, self-care, self-management and information systems. Five articles were selected.

**Results**

ICT improves people's health knowledge, promotes healthy lifestyles and changes behavior. They also reduce stress and anxiety, improve self-efficacy and self-management of chronic disease, reducing hospitalizations and the demand of health professionals.

**Conclusions**

ICTs help promote self-care for people with chronic illnesses by empowering them to make informed decisions and solve daily self-management problems. These tools are also important because they provide reliable information and can be easily queried.

### P4 How nursing students use ICT in their learning and practice

#### Ana Sofia Campos Nabais^1^, Maria do Céu Sá^1,2^

##### ^1^Escola Superior de Enfermagem de Lisboa (ESEL), Lisbon, Portugal; ^2^UI&DE, Lisboa, Portugal

###### **Correspondence:** Ana Sofia Campos Nabais (ana.nabais@esel.pt)

**Background**

Health and Information and Communication Technologies (ICT) are subjects that are present in our daily life in different domains of knowledge. The comprehension of the interaction of these areas implies recognizing health as a theme of vital importance for the human nature and the society made up by it and for it. The use of ICT’s in education has grown rapidly in the last decades, who not only changed the way we live and work but also created the need to transform the way we learn. The aim was to understand the importance attributed to ICT in the learning process among nursing students.

**Materials and Methods**

We performed individual interviews with nursing students, and the Data were treated through content analysis.

**Results**

Nursing students mainly use computer and mobile phone, they consider that these equipment facilitating the development of scientific, theoretical and theoretical-practical competences. ICT facilitates communication between people, especially between students and teachers and between health professionals and patients and helps the person with chronic illness manage their condition.

**Conclusions**

The results of the present study provide useful baseline information about which ICT devices students are using and how they are using them.

### P5 The person with dementia and the family caregiver: determinants of institutionalization. Preliminary descriptive results

#### Cátia Rosa, Graça Melo

##### Superior de Enfermagem de Lisboa (ESEL), Lisbon, Portugal

###### **Correspondence:** Cátia Rosa (catia.rosa@campus.esel.pt)

**Background**

Dementia is one of the biggest causes of disabilities in aging and providing care for the person with dementia living at home presents a complex challenge, sometimes impossible to manage. The aim was to determine the circumstances and living conditions of people with dementia, who live at home and receive formal professional care.

**Materials and Methods**

20 consecutive patients with dementia and their family caregivers, living at home. A prospective study design was used, based on RightTimePlaceCare European project protocol.

**Results**

Patients: mean age 82, 80% women; education years 3,4±2,01; 45% of the participants were diagnosed with Alzheimer´s disease, 30% marked as unknown. Family caregivers: mean age 60.8, 60% women, 70% married; education years 8,7±4,88; 35% of the family caregivers have children under 18 living with them; 50% work. 60% of the participants were at risk of institutionalization, though only 40% were in a waiting list for placement in an institutional nursing care facility. The reasons indicated by the formal caregivers were heterogeneous ranging from reasons related to the severity of the dementia and increased necessity for care to carer-burden and employment status.

**Conclusions**

These are preliminary results of an ongoing study that will be further developed for robust conclusions.

### P6 What matters for nurses to document in health information systems? The case of hypertension

#### Fernanda Bastos, Inês Cruz, Natália Machado, Alice Brito, Antónia Paiva e Silva, Alexandrina Cardoso, Paula Sousa, Paulo Parente

##### Superior Enfermagem Porto, CIDESI, Portugal

###### **Correspondence:** Fernanda Bastos (fernandabastos@esenf.pt)

**Background**

Hypertension, or high blood pressure, affects hundreds of millions of persons worldwide. What is important for nurses to document about it in a Nursing Information Systems (NIS)? The aim was to identify the data, diagnoses and interventions related to hypertension to be incorporated in an Ontology -NursingOntos, using ISO18104 and ICNP®, for any NIS.

**Materials and Methods**

A qualitative study including content analysis of the national parameterization of Portuguese nursing information system, literature review and validation of contents to be included in the ontology by a focus group.

**Results**

We have identified that several records focus on hypertension. Two categories: hypertension as an event or as a chronic disease. In first case, interventions aim at early identification and referral to the physician. In second case new data, diagnoses and interventions directed towards the person's ability to manage, control and prevent the consequences of the disease are necessary.

**Conclusions**

The NursingOntos covers documentation on the alterations in bodily process, but also suggests the inclusion of aspects related to self-management. NursingOntos do not just use ICNP® and ISO18104 but suggests the use of theoretical models of nursing.

### P7 The self-management style of clients with chronic cancer pain: influence on the medication regimen management

#### Nadine Rodrigues^1^, Fernanda Bastos^2^, Alice Brito^2^

##### ^1^Instituto Português de Oncologia do Porto, Porto, Portugal; Fernanda Bastos, F. B.; ^2^Escola Superior Enfermagem Porto (ESEP) / UNIESEP, Porto, Portugal

###### **Correspondence:** Nadine Rodrigues (nadine.p.rodrigues@gmail.com)

**Background**

In a chronic cancer pain context, the individual is challenged to incorporate a set of new behaviours to ensure symptomatic control and quality of life. Individual’s attitude towards self-care has a significant impact on the way the person manages chronic illness. The aim of the study was to identify and describe the self-management style of clients with chronic cancer pain, explore the nature of this style’s influence in the adherence to the medication regime and in the therapeutic management skills.

**Materials and Methods**

A quantitative, cross-sectional, descriptive study was conducted, based on a non-probabilistic sample of 50 individuals. A form was applied, evaluating the self-management style, therapeutic management skills and the treatment adherence.

**Results**

The self-management style was predominantly responsible (60%). Adherence levels tend to be high, but a moderate negative association was observed with the negligent score. The most responsible group and the least formally guided group presents better management skills values.

**Conclusions**

More complex regimens are prescribed to clients with better management skills. Individuals whose follow-up time in a chronic cancer pain unit is longer, have more complex drug regimens and lower peak pain intensity. This indicates that nursing therapies implemented play an important role in promoting the development of management skills.

### P8 Caring of the caregiver: characterization of the caregivers' profiles of dependent elderly in an integrated continuing care team

#### Ana Sofia Rato Santos Loureiro Pasadas^1^, Idalina Delfina Gomes^2^

##### ^1^Departamento de Psiquiatria e Saúde Mental do Hospital Beatriz-Ângelo, Loures, Portugal; ^2^Escola Superior de Enfermagem de Lisboa, Lisbon, Portugal

###### **Correspondence:** Ana Sofia Rato Santos Loureiro Pasadas (enf.ana.loureiro@gmail.com)

**Background**

In Europe, the majority of care provided to dependent elderly is carried out by the family caregivers (FC) (80%) with implications for their health and life (Teixeira et al, 2017). The Integrated Continuing Care Teams (ECCI) play a key role in promoting the care of the self (Gomes, 2016). The aim was to characterize the profile and to identify the difficulties of the FC of dependent elderly registered in a Portuguese ECCI.

**Materials and Methods**

Descriptive analytical quantitative study performed to 28 FC of dependent elderly. The collection of data was done by interviews with survey. Data were analyzed by descriptive statistics.

**Results**

The mean age of FC is around 66 years old, predominantly female (60.7%), caring of the spouse (78.6%) and their parents (32.1%). FC spend more than 12 hours per day on care (60.7%). About 64.3% of the FC relate health implications due to the caring. As difficulties, FC mentioned restrictions of social life, lack the family and professional support, physical demands of the care and financial problems.

**Conclusions**

The characterizations of FC allows intervention based on their real needs and the establishment of an Intervention Protocol to promote The Care of the Self.

**References**

Gomes, I. D. (2016). Promover o cuidado de si: parceria entre o enfermeiro e a pessoa idosa. A construção do processo de parceria num contexto de vulnerabilidade e dependência. Saarbrucken/Deutsche: Novas Edições Académicas.

Teixeira, A. R., Alves, B., Augusto B, Fonseca, C., Nogueira, J. A., Almeida, M. J. ... Nascimento, R. (2017). Medidas de intervenção junto dos cuidadores informais. Documento Enquadrador, Perspetiva Nacional e Internacional.

### P9 New method of teaching-learning as way to develop students’ clinical competences

#### Maria José Lumini, Cristina Barroso

##### Superior de Enfermagem do Porto, Porto, Portugal

###### **Correspondence:** Maria José Lumini (lumini@esenf.pt)

**Background**

The creation of tools that assist nursing students in responsible decision making has been the subject of current studies. However, in their clinical practice nursing students are often faced with some difficulties in developing their clinical skills. The study aims to identify new methods of teaching-learning that can be used to develop the students’ clinical skills in their clinical practices.

**Materials and Methods**

Retrospective, descriptive exploratory study conducted during the year 2018. The data were obtained from the analysis of 40 action plans, instrument adopt from Perez (2009) filled by the nursing students of the 3.º year of Undergraduate Degree in Nursing. Ethical approval was obtained according to the Declaration of Helsinki.

**Results**

The main goals mentioned by the students are: Communication with patients and family caregivers, knowledge about drugs, stress management, knowledge of pathophysiology, time management, instrumental skills, organizing and prioritize care, integration of knowledge in practice, improve care planning to make decisions autonomously and data collection in patient interview.

**Conclusions**

According to the findings and the actual learning environment, we agree that the creation of an informatic platform can help the students to reach their clinical goals.

### P10 eNursing intervention in people with rheumatoid arthritis

#### Ana Almeida Ribeiro^1^, Madalena Cunha^2^, Maria Adriana Henriques^3^

##### ^1^Tondela-Viseu Hospital Center, Viseu, Portugal; ^2^Polytechnic Institute of Viseu, Viseu, Portugal; ^3^Escola Superior de Enfermagem de Lisboa, Lisbon, Portugal

###### **Correspondence:** Ana Almeida Ribeiro (anaalmeidaribeiro@hotmail.com)

**Background**

People with rheumatoid arthritis (RA) present complex and multidimensional limitations, which impact their fundamental care leads to the need to focus nursing care, placing people with RA at the center of planning and decision making. Building health programs focusing in self-management improves self-efficacy and well-being, which enables the empowerment and consequent active participation of people in managing their own disease. It is essential to integrate differentiated and primary care. Through the use of eNURSING - use of technology to convey Nursing care and conduct Nursing practice – we will be able to assist health professionals and users equipped, emancipated and involved, in a partnership, aiming at the improvement of care delivery, increasing their quality of life, by cultivating proximity and care, sharing the decisions and responsibilities. The aim was to identify the typology of nursing interventions in relation to the person with RA in the Rheumatology consultation of a central Hospital Center and to implement an information system that promotes inter-institutional communicability.

**Materials and Methods**

Designing eNURSING interventions is complex and encompasses a variety of components, transporting us to the Complex Health Interventions. These will be guided by the Medical Research Council board, and the Design Science Research Methodology.

**Results**

Multi-studies and mixed methods will be used, with sequential activities being implemented such as systematic literature reviews; conducting a focus group, listening to the needs of people with RA and differentiated and primary health care professionals.

**Conclusions**

Co-production of complex interventions with the participants, to test their feasibility in promoting health literacy, self-care, quality of life and monitoring wellbeing of people with RA, will have implications in the community for the use of scientific knowledge to develop guidelines that imply the projection of good nursing practices.

### P11 Pain management in patients unable to self-report: nursing intervention

#### Magda Alcobia, Conceição Barros, Eunice Henriques

##### Superior de Enfermagem de Lisboa, Lisbon, Portugal

###### **Correspondence:** Eunice Henriques (eunice.henriques@esel.pt)

**Background**

Pain control is a patient´s right, a nurse´s obligation and a fundamental step for the effective humanization of care. Studies confirm that despite regularly receiving analgesia, the vast majority of critically ill patients continue to suffer from pain, resulting in prolonged ICU stay, the need for invasive mechanical ventilation and increased incidence of sequelae. The aims of the study are (1) to understand how pain diagnosis is performed with mechanically ventilated patients who are unable to self-report; (2) to analyse factors that influence both the diagnosis of pain and the decision making processes to control it; and (3) to identify evidence-based recommendations for effective pain management in ventilated patients unable to self-report.

**Materials and Methods**

An integrative literature review using Whittemore and Knafl’s integrative review method will be performed through MEDLINE, CINAHL and the Cochrane Library with articles published between January the 1st of 2012 and January the 31st of 2019. The review includes studies with patients unable to self-report, critically ill and mechanically ventilated.

**Results**

Results will be reported.

**Conclusions**

Nurses tend to underestimate patient´s pain and to misinterpret some behavioural manifestations. Frequent and structured pain assessment, through valid and reliable tools, combined with the use of pain management protocols, reduces the incidence of false-based routines and increases the effectiveness of pain management.

### P12 Antenatal breastfeeding educational program: its impact on breastfeeding knowledge, attitudes, self-efficacy and perceived barriers among pregnant women in Greece

#### Maria Iliadou^1,2^, Panagiota Kalatzi^1^, Styliani Tziaferi^1^

##### ^1^Department of Nursing, University of Peloponnese, Sparti, Greece; ^2^Department of Midwifery, University of West Attica, Athens, Greece

###### **Correspondence:** Styliani Tziaferi (stylianitziaferi216@gmail.com)

**Background**

The goal of antenatal breastfeeding education is to influence breastfeeding indicators. In Greece, a country with low breastfeeding rates, the impact of this kind of intervention on the modifiable factors of breastfeeding knowledge, attitudes, self-efficacy and perceived barriers has not been yet evaluated. The primary outcome was to evaluate the impact of an educational intervention on the breastfeeding indicators.

**Materials and Methods**

This was a quasi-experimental study that employed a pre-post design, with an intervention (n=103 pregnant women) and a control group (n=100 pregnant women), at a tertiary hospital in Athens- Greece, during May 2016 - January 2017. Data were collected by using various scales.

**Results**

Pre intervention there were no significant differences between control and intervention group in any of the scales. Post intervention, women in the intervention group had a more positive attitude towards breastfeeding (73.5% versus 66.1%, p<0.001), greater knowledge (14.6% versus 13.1%, p<0.001) and increased breastfeeding self-efficacy (51.4% versus 45.6%, p<0.001) compared to the control group. Furthermore, they had significantly less perceived barriers regarding breastfeeding (27.4% versus 31.0%, p<0.001).

**Conclusions**

Antenatal breastfeeding education has a remarkable positive impact on breastfeeding indicators compared to routine care.

**References**

Mora ADL, Russell DW, Dungy CI, Losch M, & Dusdieker L. The Iowa infant feeding attitude scale: analysis of reliability and validity. Journal of Applied Social Psychology 1999;29 p: 2362-2380.

Dennis CL, and Faux S. Development and psychometric testing of the Breastfeeding Self-Efficacy Scale. Research in Nursing and Health 1999;22 p: 399-409.

Casal CS, Lei A, Young SL, & Tuthill EL. A Critical Review of Instruments Measuring Breastfeeding Attitudes, Knowledge, and Social Support. Journal of Human Lactation 2016;doi:0890334416677029.

### P13 Ageing immunosenescence and vaccine efficacy

#### Panagiota Kalatzi^1^, Maria Iliadou^2^, Styliani Tziaferi^2^

##### ^1^Department of Nursing, University of Peloponnese, Sparti, Greece; ^2^Department of Midwifery, University of West Attica, Athens, Greece

###### **Correspondence:** Styliani Tziaferi (stylianitziaferi216@gmail.com)

**Background**

The age- dependent decrease in immunological competence results in greater susceptibility to infection and reduced responses to vaccination. The present study aims to increase the understanding of immunosenescence and investigate strategies to overcome vaccine ineffectiveness.

**Materials and Methods**

Literature review in PubMed, Cinahl and Google Scholar was conducted between February and March 2018 with the following keywords: immunosenescence, vaccination, elderly and efficacy. English written reports published in or after 2010 that examined the impact of age related immune deficiency and vaccine failure were included.

**Results**

Age related immunological alterations include reduction in natural killer T- cell and B- cell cytotoxicity and disturbances in macrophage- derived cytokine release. Thymic atrophy, reduced output of anergic memory cells, deficiencies in the cytokine production, uncertain antigen presentation and coinfection with persistent viruses results in further declination of the cellular immunity among older adults. New formulations such as booster vaccinations, different immunization routes, high dose vaccines and the use of adjuvants are approaches that are under investigation and may improve the efficacy and effectiveness of immunization.

**Conclusions**

Understanding mechanisms of immunological aging and designing more effective vaccines may strengthen infectious diseases prevention among older adults. This study has a great relevance for public health nursing science across the Europe.

**References**

Boraschi D, Italiani P. Immunosenescence and vaccine failure in the elderly: Strategies for improving response. Immunology Letters. 2014;162(1):346-353.

Fuentes E, Fuentes M, Alarcon M, Palomo I. Immune System Dysfunction in the Elderly. An Acad Bras Cienc. 2017;89(1):285-299.

Loukov D, Naidoo A, Bowdish D. Immunosenescence: implications for vaccination programs in the elderly. Vaccine: Development and Therapy. 2015;5:17-29.

### P14 Results of Data Collection from the Limoxis System for the Control and Management System of Hospital Acquired Infections

#### Filippos Gozadinos^1^, Styliani Tziaferi^2^, Athina Lazakidou^1^

##### ^1^University of Peloponnese, Department of Economics, Tripolis, Greece; ^2^University of Peloponnese, Department of Nursing, Sparta, Greece

###### **Correspondence**: Styliani Tziaferi (stylianitziaferi216@gmail.com)

**Background**

Limoxis can be utilized in the Hospital Units as a useful tool to collect all necessary data for Hospital Acquired Infections. Participants were 10 Hospital Units of the 6th Health District and 1 Public Hospital Unit of Attica.

**Materials and Methods**

A total of 1330 cases of notifiable-disease and bacteraemia reporting forms from period 2010-2016 of 10 Hospital Units of the 6th Health District and Public Hospital Unit of Attica were digitized for epidemiological study and applied to the newly-developed information system.

**Results**

The total of 42.3% of all patients suffered from Hospital Acquired Infections caused by pathogen Acinetobacter and 38.8% from pathogen Klebsiella. Depending on the categories of HAIs, bloodstream ones prevail against pneumonia (34.4%) and urinary track ones (22.3%). Depending on the types of bacteraemia, primary bacteraemia prevails with 61.5%. Most patients have had a hospital stay of more than 28 days.

**Conclusions**

The Limoxis Information System enables data entry and electronic storage, which takes less time than printed forms and also allows fast access to stored data. Finally, there are important options such as statistical analysis, provides the antibiotics under surveillance, provides also a reliable estimate of antibiotic consumption and the geographical mapping of data useful for Healthcare Professionals and Hospital Administration.

### P15 Cultural Adaptation in Greek language and Validation of the instrument «Bullying in Nursing Education Questionnaire (BNEQ)» to university nursing students

#### Kalafati Maria^1^, Tziaferi Styliani^2^, Nieri Alexandra Stauvroula^1^, Dede Maria Niki^2^, Karanikola Maria^3^, Mpouzika Meropi^3^, Lemonidou Chryssoula^1^

##### ^1^Faculty of Nursing, National and Kapodistrian University of Athens, Athens, Greece; ^2^Faculty of Nursing, University of Peloponnese, Peloponnese, Greece; ^3^Faculty of Nursing, Cyprus University of Technology

###### **Correspondence:** Tziaferi Styliani (stylianitziaferi216@gmail.com)

**Background**

Bullying is a phenomenon that observed in nursing students and related to negative effects on physical and mental health. The aim of the study was validation and cultural adaptation of the questionnaire "Bullying in Nursing Education Questionnaire (BNEQ)" in Greek university Students of Nursing.

**Materials and Methods**

A pilot correlational study was performed in a sample of 25 nursing students. The BNEQ was translated from English into Greek, and backward, and its cultural adaptation took place. Cronbach's αand test-retest reliability (Wilcoxon, Spearman) and x2 test were applied at statistical significance α = 0.05.

**Results**

The Cronbach alpha coefficient was equal to 0.96. All questions were significantly correlated (p <0.05), with the exception of the questions "Yelling or shouting in rage to you from ....", "Inappropriate, nasty, rude or hostile behavior to you from ...", "Spreading of malicious rumors or gossip to you from ... ", "Cursing or swearing to you from…","Negative or disparaging remarks about becoming a nurse..."and "Being ignored or physically isolated... ". The Wilcoxon test showed that there was no statistically significant difference (p> 0.05), with the exception of the questions "Yelling or shouting in rage to you from ....", "Inappropriate, nasty, rude or hostile behavior to you from ...", and "Negative or disparaging remarks about becoming a nurse...".

**Conclusions**

The pilot data support the reliability and validity of the BNEQ scale

.

### P16 Development and feasibility of a psychoeducational intervention to promote adolescents' mental health literacy in a school context

#### Tânia Morgado^1^, Luís Loureiro^2^, Maria Antonia Rebelo Botelho^3^

##### ^1^Pedopsiquiatria, Centro Hospitalar e Universitário de Coimbra, EPE, Coimbra, Portugal; ^2^ Escola Superior de Enfermagem de Coimbra, Coimbra, Portugal; ^3^ Escola Superior de Enfermagem de Lisboa, Lisbon, Portugal

###### **Correspondence:** Tânia Morgado (tmorgado@gmail.com)

**Background**

Mental Health Literacy was defined as knowledge and beliefs about mental disorders that aid in its recognition, management or prevention. Portugal is the European country with the highest prevalence of mental illness (22.9%) and anxiety disorders are the most common (16.5%). Portugal School Health Program 2015 evidences the promotion of health literacy and anxiety as areas of intervention in adolescence. The aim was to identify evidence about adolescents’ anxiety mental health literacy in school context; Design a psychoeducational intervention; Validate the content of that intervention; Evaluate its feasibility.

**Materials and Methods**

Medical Research Council framework for development and evaluation of complex interventions using mixed methods throughout development and feasibility stages: 1) systematic reviews; 2) health/education professionals’ and adolescents’ focus group; 3) experts’ e-Delphi; 4) feasibility study; 5) pilot study.

**Results**

The psychoeducational intervention has different methods and pedagogical techniques, consists of 4 sessions of 90 minutes each. We observed clinically and statistically significant impact.

**Conclusions**

This psychoeducational intervention increased adolescents’ anxiety mental health literacy and allow them to access, understand and use information on prevention, recognition and management of anxiety. This study has a high relevance for nursing science in Portugal and Europe. Trial Registration: ClinicalTrials.gov ID: NCT03872817.

### P17 Mapping antenatal care policy for low-risk pregnant women in Portugal and comparable countries: A scoping review

#### Andreia S Gonçalves^1^, Isabel M Ferreira^1^, Ana Paula Prata^2^, Christine McCourt^3^, António Correia de Campos^4^

##### ^1^ASG and IMF, Instituto de Ciências Biomédicas Abel Salazar, University of Porto; ^2^APP, Escola Superior de Enfermagem do Porto, Porto, Portugal; ^3^CMc, School of Health Sciences, City University London, London, United Kingdom; ^4^ACC, Conselho Económico Social, Lisboa, Portugal

###### **Correspondence:** Andreia S Gonçalves (avasgoncalves@gmail.com)

**Background**

It is recognised that in high-income countries more can be done so that women can experience a safe and positive pregnancy. Midwives were identified as the key practitioners to provide this when compared to other models of care1. In Portugal antenatal care of low-risk healthy pregnant women is provided by family-doctors despite having available nurse-midwives who are specially trained to maintain “normality”2. The aim of the study was to map antenatal care policy in high income countries comparable to Portugal, with the ultimate goal of informing future policy and identify the viability of the adoption of a midwifery-led-care model to the Portuguese context.

**Materials and Methods**

A search was conducted in MEDLINE, CINAHL, Academic Search Complete, Web of Science and Scopus. A hand search of grey literature of published policy documents followed and for the countries whose policies were not available key persons were contacted. Search results were exported and data extracted using charting forms. Data will be synthesised using narrative description.

**Results**

The authors are still working on the data but preliminary results show a wide variability in nature, content and range of the policies

**References**

Sandall, J., Soltani, H., Gates, S., Shennan, A., & Devane, D. (2016). Midwife-led continuity models versus other models of care for childbearing women. Cochrane Database Syst Rev, 4(9), Cd004667. https://doi.org/10.1002/14651858.CD004667.pub5

Publication charges for this supplement were funded by the conference.

